# An interferon-like small chemical compound CDM-3008 suppresses hepatitis B virus through induction of interferon-stimulated genes

**DOI:** 10.1371/journal.pone.0216139

**Published:** 2019-06-12

**Authors:** Yutaka Furutani, Mariko Toguchi, Yumi Shiozaki-Sato, Xian-Yang Qin, Etsuko Ebisui, Shoko Higuchi, Masayuki Sudoh, Harukazu Suzuki, Nobuaki Takahashi, Koichi Watashi, Takaji Wakita, Hideaki Kakeya, Soichi Kojima

**Affiliations:** 1 Liver Cancer Prevention Research Unit, RIKEN Center for Integrative Medical Sciences, Wako, Saitama, Japan; 2 Laboratory for Cellular Function Conversion Technology, RIKEN Center for Integrative Medical Sciences, Tsurumi-ku, Yokohama, Kanagawa, Japan; 3 Department of System Chemotherapy and Molecular Sciences, Division of Bioinformatics and Chemical Genomics, Graduate School of Pharmaceutical Sciences, Kyoto University, Sakyo-ku, Kyoto, Japan; 4 Department of Virology II, National Institute of Infectious Diseases, Tokyo, Japan; Konkuk University, REPUBLIC OF KOREA

## Abstract

Oral administration of nucleotide analogues and injection of interferon-α (IFNα) are used to achieve immediate suppression in replication of hepatitis B virus (HBV). Nucleotide analogs and IFNα inhibit viral polymerase activity and cause long-term eradication of the virus at least in part through removing covalently closed circular DNA (cccDNA) via induction of the APOBEC3 deaminases family of molecules, respectively. This study aimed to explore whether the orally administrable low molecular weight agent CDM-3008 (RO8191), which mimics IFNα through the binding to IFNα/β receptor 2 (IFNAR2) and the activation of the JAK/STAT pathway, can suppress HBV replication and reduce cccDNA levels. In primary cultured human hepatocytes, HBV DNA levels were decreased after CDM-3008-treatment in a dose-dependent manner with a half-maximal inhibitory concentration (IC_50_) value of 0.1 μM, and this was accompanied by significant reductions in cellular cccDNA levels, both HBeAg and HBsAg levels in the cell culture medium. Using a microarray we comprehensively analyzed and compared changes in gene (mRNA) expression in CDM-3008- and IFNα-treated primary cultured human hepatocytes. As reported previously, CDM-3008 mimicked the induction of genes that participate in the interferon signaling pathway. OAS1 and ISG20 mRNA expression was similarly enhanced by both CDM-3008 and IFNα. Thus, CDM-3008 could suppress pgRNA expression to show anti-HBV activity. APOBEC3F and 3G mRNA expression was also induced by CDM-3008 and IFNα treatments, suggesting that cccDNA could be degraded through induced APOBEC3 family proteins. We identified the genes whose expression was specifically enhanced in CDM-3008-treated cells compared to IFNα-treated cells. The expression of SOCS1, SOCS2, SOCS3, and CISH, which inhibit STAT activation, was enhanced in CDM-3008-treated cells suggesting that a feedback inhibition of the JAK/STAT pathway was enhanced in CDM-3008-treated cells compared to IFNα-treated cells. In addition, CDM-3008 showed an additive effect with a clinically-used nucleoside entecavir on inhibition of HBV replication. In summary, CDM-3008 showed anti-HBV activity through activation of the JAK/STAT pathway, inducing the expression of interferon-stimulated genes (ISGs), with greater feedback inhibition than IFNα.

## Introduction

More than 200 million people are chronically infected with hepatitis B virus (HBV) worldwide [1–3]. HBV is associated with the development of hepatocellular carcinoma (HCC) through progression of cirrhosis [4–6]. Thus, drug development for the elimination of HBV is urgently need.

HBV virions contain partially double-stranded relaxed circular DNA (rcDNA). After internalization into hepatocytes, rcDNA is converted into covalently closed circular DNA (cccDNA) using an intracellular DNA repair mechanism, and cccDNA then starts transcription of pregenomic RNA (pgRNA) and mRNAs for surface antigen, capsid, polymerase, and X protein. pgRNA is reverse-transcribed by polymerase in the capsid, and HBV virions are then released after being coated with surface antigen [7].

Nucleotide analogs are mainly used to suppress HBV replication, but these drugs cannot completely remove cccDNA and pose a risk of drug resistance [8–10]. Interferon-α (IFNα) treatment induces interferon-stimulated genes (ISGs) through activation of the janus kinase/signal transducers and activators of transcription (JAK/STAT) signaling pathway and shows anti-HBV activity mainly through RNase L activity for suppression of pgRNA [11–13], although IFNα is associated with several side effects and induces hepatitis B e antigen (HBeAg) seroconversion in approximately 30% of chronically infected HBV patients [14,15]. Proteins of the apolipoprotein B mRNA-editing enzyme catalytic polypeptide–like 3 (APOBEC3) family have recently been reported to trigger the degradation of cccDNA through their deaminase activity stimulated by IFNα, IFNγ, lymphotoxinβ, and TNFα [16,17]. Because cccDNA can be degraded after IFN administration, development of IFN-based drugs is important for complete cure in case of HBV infection. Cotreatment with an orally available IFN-based drug and nucleotides/nucleosides could effectively suppress HBV. Poly(I:C) and GS9620, Toll-like receptor agonists, have been reported to exert anti-HBV activity through IFN expression [18,19]. To directly induce ISGs and reduce side effects, the development of IFN-based small compounds is desirable.

As the first step to improve anti-HBV activity and reduce the side effects of IFNα [20], we focused on a small chemical compound, RO8191, which functions as an IFNα/β receptor 2 (IFNAR2) agonist. RO8191 binds to IFNAR2 and it induces ISG expression through the JAK/STAT signaling pathway, and showing anti-hepatitis C virus activity [21]. RO8191 can be administrated orally and produced at a low cost and thus has an advantage over IFNα, which must be injected [21]. In this study, we discovered that RO8191 has anti-HBV activity and modulates cccDNA levels through interferon-like activity. Thus, we named RO8191 as a cccDNA modulator (CDM) and numbered it 3008. To further understand the molecular basis of the anti-HBV activity of CDM-3008, we compared the activities of CDM-3008 and IFNα on gene expression pattern in human primary hepatocytes using a microarray.

## Materials and methods

### Culture of primary human hepatocytes (PXB cells) isolated from chimeric mice with humanized livers

PXB cells were purchased from PhoenixBio and cultured on collagen type I-coated 96 well or 24 well plates in hepatocyte clonal growth medium (dHCGM) under 37°C and 5% CO_2_ as previously described [22].

### Assay of anti-HBV activity using PXB cells

PXB cells were infected with 3 genome equivalents per cell of HBV C_AT [23], and the medium was exchanged every 3 or 4 days. After 28 days of infection, CDM-3008 was serially diluted in DMSO from 0.03–10 μM and mixed with dHCGM, and the cells were then treated with a final concentration of 0.0003–100 μM CDM-3008 for 7 days.

After infection and treatments, the cell culture medium was collected, and cell viability was measured using the RealTime-Glo MT Cell Viability Assay (Promega). Cellular DNA was then purified using the Agencourt DNAdvance System (Beckman Coulter).

### MT cell viability assay

After treatment with compounds, cell viability was measured using the RealTIme-Glo MT Cell Viability Assay (Promega) according to the manufacturer’s instructions. The cells were incubated with 95 μl of cell culture medium, 2.5 μl of MT Cell Viability Substrate, and 2.5 μl of NanoLuc Enzyme under 5% CO_2_ at 37 °C for 60 min. To detect luminescence, 50 μl of the medium was transferred to 96-well white plates (3912, Costar), and luminescence intensity was measured using a multi-mode plate reader (EnSight, PerkinElmer).

### XTT assay

After treatment with compounds, cell viability was measured using the Cell Proliferation Kit II (Roche) according to the manufacturer’s instructions. The cells were incubated with 100 μl of cell culture medium and 50 μl of XTT reagent (Cell Proliferation Kit II, Roche) under 5% CO_2_ at 37 °C for 45 min. The absorbance at 492 nm was then measured using a multi-mode plate reader (EnSight, PerkinElmer).

### Knockdown of IFNAR2

IFNAR2 siRNA (siGENOME SMART pool Human IFNAR2, M-015411-00, Dharmacon) or control siRNA (siGENOME Control pool Nontargeting #1, D-001206-13-05, Dharmacon) were mixed with Dharma FECT 4 in OPTI-MEM (Thermo), and HCV replicon cells were then mixed with the siRNA and plated in 96 well plates. After 2 days of incubation, the cells were treated with 30 μM CDM-3008 for 8 h. Total RNA was purified from the cells using Agencourt RNAdvance (Beckman).

### IFNβ-treatment after incubation with CDM-3008 or IFNα

PXB cells were treated with 30 μM CDM-3008 or 10 ng/ml PEGylated IFNα2a (Pegasys, Chugai pharmaceutical) for 24 h followed by treatment with 10 ng/ml recombinant IFNβ (Wako) for 4 h. Total RNA was purified from the cells using Agencourt RNAdvance Cell v2 (Beckman).

### DNA purification from cultured cells

After treatment with compounds, DNA was automatically purified using the Agencourt DNAdvance Genomic DNA Isolation Kit and an automatic dispenser (Biomek i5 or NXp, Beckman Coulter) according to the manufacturer’s instructions. Briefly, the cells were washed 3 times with PBS and incubated with 200 μl of Lysis buffer containing 50 mM dithiothreitol (DTT) and proteinase K at 55 °C for 1 h with constant agitation. The lysates were transferred to 96-well 1.2-ml deep well storage plates (Thermo, AB1127) and mixed with 100 μl of Bind1 buffer, and then the 170 μl of Bind2 buffer was then added, and the samples were again mixed. After collection of the DNA-binding magnetic beads, the beads were washed twice with 340 μl of 70% ethanol. DNA was eluted with 200 μl of MilliQ water.

### DNA purification from cell culture medium

After collection of the cell culture medium, the DNA present in the medium was automatically purified using Agencourt Genfind v2 and an automatic dispenser (Biomek i5 or NXp, Beckman Coulter) according to the manufacturer’s instructions. Briefly, 50 μl of cell culture medium was mixed with 200 μl of Lysis buffer containing Proteinase K in a 96-well 1.2-ml deep-well storage plate (Thermo, AB1127); the mixture was then incubated at 37 °C for 3 h with constant agitation. After centrifugation at 1,000xg for 1 min, 150 μl of Binding buffer was added to the lysates and mixed. The DNA-binding magnetic beads were washed twice with 400 μl of Wash buffer 1 and twice with 250 μl of Wash buffer 2. DNA was eluted with 50 μl of MilliQ water.

### RNA purification from cultured cells

#### RNeasy mini kit (Qiagen) for RNA purification from cells cultured in 24-well plates

After washing the cells with PBS 3 times, RNA purification was performed using the RNeasy mini kit (Qiagen) according to the manufacturer’s instructions.

#### Agencourt RNAdvance Cell v2 (Beckman Coulter) for RNA purification from cells cultured in 96-well plates

RNA purification was automatically performed using Agencourt RNAdvance Cell v2 and an automatic dispenser (Biomek i5 or NXp, Beckman Colter) according to the manufacturer’s instructions. The cells were washed 3 times with PBS and then incubated with 63 μl of Lysis buffer containing proteinase K at 25 °C for 30 min and stored at -80 °C. After thawing and centrifugation at 1,000xg for 15 s, 175 μl of Binding buffer solution was mixed with the lysates in a 96-well 1.2-ml deep-well storage plate (Thermo, AB1127). The RNA-binding magnetic beads were washed with 200 μl of Wash buffer followed by 200 μl of 70% ethanol and then incubated with 5 U DNaseI (Nippon gene) at 25 °C for 15 min. The beads were then washed once with 138 μl of Wash buffer and twice with 200 μl of 70% ethanol. RNA was eluted with 40 μl of MilliQ water (RNase and DNase free).

### T5 exonuclease treatment

T5 exonuclease treatment was performed according to the method described previously [24]. Briefly, 89 μl of purified DNA using an Agencourt DNAdvance Genomic DNA Isolation Kit was treated with 10 U of T5 exonuclease (New England Biolabs) at 37 °C for 1 h, and T5 exonuclease was inactivated at 70 °C for 20 min. DNA was ethanol-precipitated and redissolved in 5 μl of 10 mM Tris/HCl pH 7.5 containing 1 mM EDTA.

### qPCR analysis of HBV DNA and cccDNA

HBV DNA and cccDNA copy numbers were determined using TaqMan Gene Expression Master Mix (Thermo Fisher) or Probe qPCR Mix (Takara), specific primers, and probes in a quantitative PCR system (Light Cycler96, Roche or CFX-96, Bio-rad). The specific primers and probes used for detection of HBV DNA were shown (forward primer: 5’-ACTCACCAACCTCCTGTCCT-3’, reverse primer: 5’-GACAAACGGGCAACATACCT-3’, and probe: 5’-[FAM] TATCGCTGGATGTGTCTGCGGCGT[TAM]-3’). The specific primers and probes used for detection of cccDNA were developed by Qu et al. [24] and designed for HBV C_AT (forward primer: 5’-GTGGCTATCCTGCCTTAAT-3’, reverse primer: 5’-CAGAGCTGAGGCGGTGTC-3’, and probe: 5’-[FAM] AGTTGGCGAGAAAGTGAAAGCCTGC [TAM]-3’).

### qPCR analysis of ISGs induction

ISGs mRNA expression levels were measured by qPCR using specific primers or sets of specific primers and probe. RNA was reverse transcribed using PrimeScript RT Master Mix (Takara) to synthesize cDNA. IFNAR1 and IFNAR2 mRNA levels were measured using sets of specific primers and probes (Hs01066116 m1 IFNAR1 and Hs01022059 m1 IFNAR2, Applied Biosystems) and Probe qPCR mix (Takara) in a Light Cycler 96 qPCR system (Roche). STAT2, ISG15, ISG20, USP18, SOCS1, SOCS2, SOCS3, CISH, APOBEC3F, APOBEC3G, SLPI, WFDC2, and GAPDH mRNA expression levels were measured using specific primers and TB Green Premix Ex Taq II (Takara) in a Light Cycler 96 qPCR system (Roche). The specific primers used in this study were ISG15 (forward primer: 5’ TCCTGGTGAGGAATAACAAGGG-3’, reverse primer: 5’- GTCAGCCAGAACAGGTCGTC-3’), ISG20 (forward primer: 5’-TAGCCGCTCATGTCCTCTTT-3’, reverse primer: 5’-TGAGGGAGAGATCACCGATT-3’), USP18 (forward primer: 5’- CCTGAGGCAAATCTGTCAGTC-3’, OAS1 (forward primer: 5’- TCCGTGAAGTTTGAGGTCCA-3’, reverse primer: 5’- ATCAAAGGCAGGCAGCACAT-3’), reverse primer: 5’- CGAACACCTGAATCAAGGAGTTA-3’), SOCS1 (forward primer: 5’-GCCCCTTCTGTAGGATGGTA-3’, reverse primer: 5’-CTGCTGTGGAGACTGCATTG-3’), SOCS2 (forward primer: 5’-ATGACCCTGCGGTGCCT-3’, reverse primer: 5’-AAAGTTCCTTCTGGTGCCTCT-3’), STAT2 (forward primer: 5’-CATACTAGGGACGGGAAGTCG-3’, reverse primer: 5’-ATTCTGCAGCATTTCCCACT-3’), SOCS3 (forward primer: 5’-CTTCGACTGCGTGCTCAA-3’, reverse primer: 5’-GTAGGTGGCGAGGGGAAG-3’), STAT2 (forward primer: 5’-CATACTAGGGACGGGAAGTCG-3’, reverse primer: 5’-ATTCTGCAGCATTTCCCACT-3’), CISH (forward primer: 5’- TTCTGCACTCAGGGAGGACT-3’, reverse primer: 5’-CAGGAGGAAGGAACTTGCTG-3’), APOBEC3F (forward primer: 5’-CCGTTTGGACGCAAAGAT-3’, reverse primer: 5’-CCAGGTGATCTGGAAACACTT-3’), APOBEC3G (forward primer: 5’-CCGAGGACCCGAAGGTTAC-3’, reverse primer: 5’-TCCAACAGTGCTGAAATTCG-3’), SLPI (forward primer: 5’-GAGATGTTGTCCTGACACTTGTG-3’, reverse primer: 5’-AGGCTTCCTCCTTGTTGGGT-3’), WFDC2 (forward primer: 5’-CGGCTTCACCCTAGTCTCAG-3’, reverse primer: 5’-CCTCCTTATCATTGGGCAGA-3’) and GAPDH (forward primer: 5’-CAATGACCCCTTCATTGACC-3’, reverse primer: 5’-GACAAGCTTCCCGTTCTCAG-3’).

### Measurement of hepatitis B antigens

Hepatitis B s antigen (HBsAg) and hepatitis B e antigen (HBeAg) levels in the cell culture medium were measured using Enzygnost HBsAg 6.0 and Enzygnost HBe monoclonal (Siemens), respectively, according to the manufacturer’s instructions.

### 3D-PCR detection of APOBEC3 activity

PXB cells were infected with 3 genome equivalents per cell of HBV C_AT [23], and the medium was exchanged every 3 or 4 days. After 28 days of infection, the cells were treated with 100 μM CDM-3008 and 10,000 IU/ml IFNα (Sumitomo Dainippon Pharma) in dHCGM for 7 days. After infection and treatments, the cellular DNA was then purified using the Agencourt DNAdvance System (Beckman Coulter). 3D-PCR was performed according to standard hepatitis B virus methods and protocols [25]. Briefly, cccDNA was amplified from the purified DNA by PCR (PCR Thermal Cycler Dice Touch, Takara) using specific primers designed for HBV C_AT sequence (forward primer: 5’-AGAGCTGAGGCGGTGTCGAG-3’, reverse primer: 5’-ACCTATTGATTGGAAAGTATGT-3’) and KOD One PCR Master Mix (Toyobo). The HBx region was amplified by nested PCR using specific primers (forward primer: 5’-ATGGCTGCTARGCTGTGCTGCCAA-3’, reverse primer: 5’-AAGTGCACACGGTYYGGCAGAT-3’) and KOD One PCR Master Mix (Toyobo) with gradient of denaturation temperature (80–92 °C). The nested PCR products were separated by 2% agarose gel electrophoresis, visualized with ethidium bromide, subcloned using the Zero Blunt TOPO PCR cloning kit (Thermo Fisher), and sequenced using the BigDye Terminator v3.1 sequencing kit (Thermo Fisher).

### Microarray analysis

PXB cells cultured in 24-well plates were not infected or were infected with 3 genome equivalents per cell of HBV C_AT. After 30 days of infection, the cells were treated with 30 μM CDM-3008 and 10 ng/ml PEGylated IFNα2a (Pegasys, Chugai pharmaceutical) for 4 and 8 h (n = 3). Following treatments, total RNA was purified from the cells using an RNeasy mini kit (Qiagen). The amount of total RNA was measured using a NanoDrop ND-1000 (NanoDrop Technologies). Microarray analysis was performed using the Illumina platform. Five hundred nanograms of total RNA was used for first-stranded cDNA and second-stranded cRNA preparation with the Illumina TotalPrep RNA Amplification Kit (Ambion, USA). The converted cRNA was then labeled and hybridized using the Human HT-12 v4 Expression BeadChip Kit according to the manufacturer’s instruction. Experimental triplicate samples were prepared for microarray analysis, and the data were processed with a package from Bioconductor (lumi) using a free software environment, R (http://www.r-project.org/). The raw and normalized data were uploaded to NCBI Gene Expression Omnibus (GSE126090).

### Knowledge-based pathway analysis

To explore the biological interpretation of the transcriptome data, the canonical pathway was identified using knowledge-based functional analysis software from Ingenuity Pathways Analysis (IPA; Ingenuity Systems).

### Statistical analysis

Student’s *t*-test was performed to compare mean using Microsoft Excel 2013.

## Results

**Fig 1:**
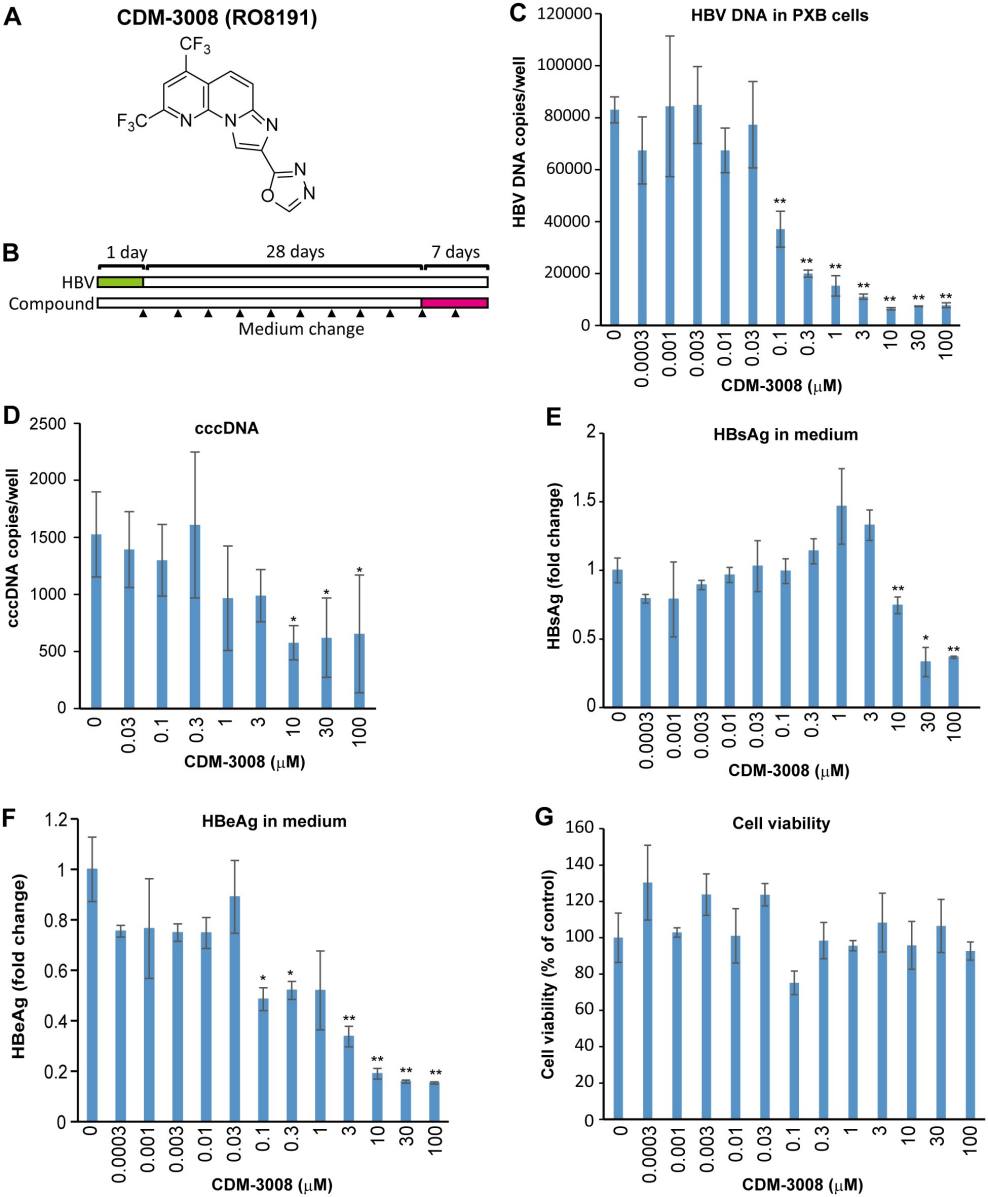
Anti-HBV activity of CDM-3008 in primary cultured human hepatocytes. (A) The chemical structure of CDM-3008. (B) The schematic experimental design of the anti-HBV activity analysis. Primary cultured human hepatocytes (PXB cells) were infected with HBV genotype C for 1day (green) and cultured for 28 days, and the cells were then treated with 0–100 μM CDM-3008 for 7 days (magenta). The black triangles show the time points of medium changes. (C) Measurement of HBV DNA copies in PXB cells after 7 days of CDM-3008 treatment. HBV DNA was decreased with 0.1–100 μM CDM-3008 in a dose-dependent manner. (D) Measurement of cccDNA copies after T5 exonuclease treatment. cccDNA was significantly decreased with 10–100 μM CDM-3008. Error bars indicate S.D. in D (n = 3). *, p < 0.05 (one-tailed *t*-test) in D. (E) HBsAg levels are shown as fold changes. HBsAg levels were significantly decrease with 10–100 μM CDM-3008. (F) HBeAg levels are shown as fold changes. HBeAg levels were significantly decreased with 0.1–100 μM CDM-3008. (G) Cell viabilities were shown as % of control DMSO. Error bars indicate S.D. in C, E-G (n = 3). *, p < 0.05 and **, p < 0.01 (two-tailed *t*-test) in C, E-G.

Anti-hepatitis C virus (HCV) activity of CDM-3008 was previously reported by Konishi et al. [21], but anti-HBV activity of CDM-3008 was remain unknown. To demonstrate the anti-HBV activity of CDM-3008 whose chemical structure is shown in Fig 1A, primary cultured human hepatocytes (PXB cells) were infected with HBV genotype C and cultured for 28 days. Then, the cells were treated with serially diluted CDM-3008 for 7 days (Fig 1B). HBV DNA was decreased in the cells upon CDM-3008 treatment in a dose-dependent manner (Fig 1C). The IC_50_ for inhibition of cellular HBV DNA was calculated as 0.1 μM. cccDNA in the cells, HBeAg and HBsAg in the medium were also significantly decreased with CDM-3008 treatment (Fig 1D–1F) without showing dose-dependent cytotoxicity (Fig 1G).

**Fig 2:**
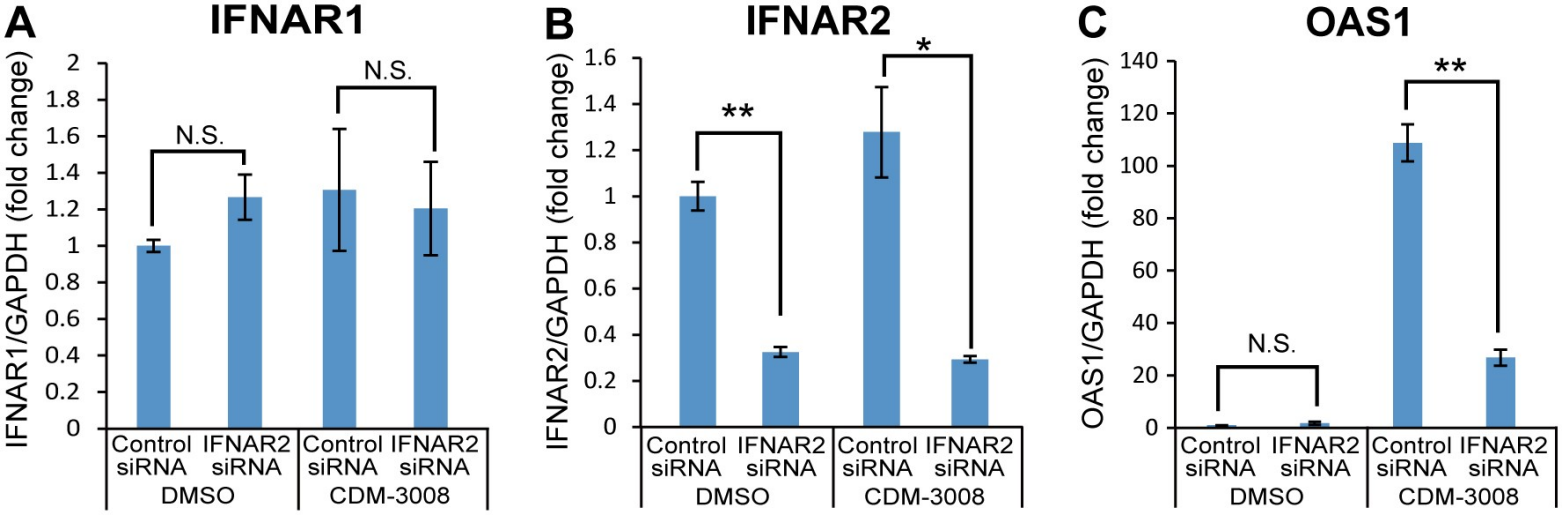
Suppression of ISGs expression after knockdown of IFNAR2. HCV replicon cells were treated with IFNAR2 siRNA for 2 days and incubated with CDM-3008 for 8 h. (A) IFNAR1 expression after control and IFNAR2 siRNA treatment. IFNAR1 expression was not suppressed by IFNAR2 siRNA treatment. (B) IFNAR2 expression after control and IFNAR2 siRNA treatment. IFNAR2 mRNA expression was suppressed by treatment with IFNAR2 siRNA but not by treatment with control siRNA. (C) OAS1 expression after control and IFNAR2 siRNA treatment. OAS1 mRNA expression was suppressed by treatment with IFNAR2 siRNA but not by treatment with control siRNA. The error bars in A-C indicate S.D. (n = 3). *, p < 0.05; **, p < 0.01 (two-tailed *t*-test) in A-C.

Konishi et al. reported that CDM-3008 induced ISGs expression and suppressed HCV replicon through IFNAR2 [21]. To show that anti-HBV activity was mainly regulated by ISGs expression through IFNAR2, we analyzed 2'-5'-oligoadenylate synthase 1 (OAS1) expression after knockdown of IFNAR2. However, knockdown of IFNAR2 in PXB cells did not work by unknown reason. Thus, knockdown of IFNAR2 was performed in HCV replicon cells instead of in PXB cells. Induction of OAS1 was suppressed by knockdown of IFNAR2 (Fig 2A–2C). Thus, CDM-3008 induced ISGs expression through IFNAR2 activation.

**Fig 3:**
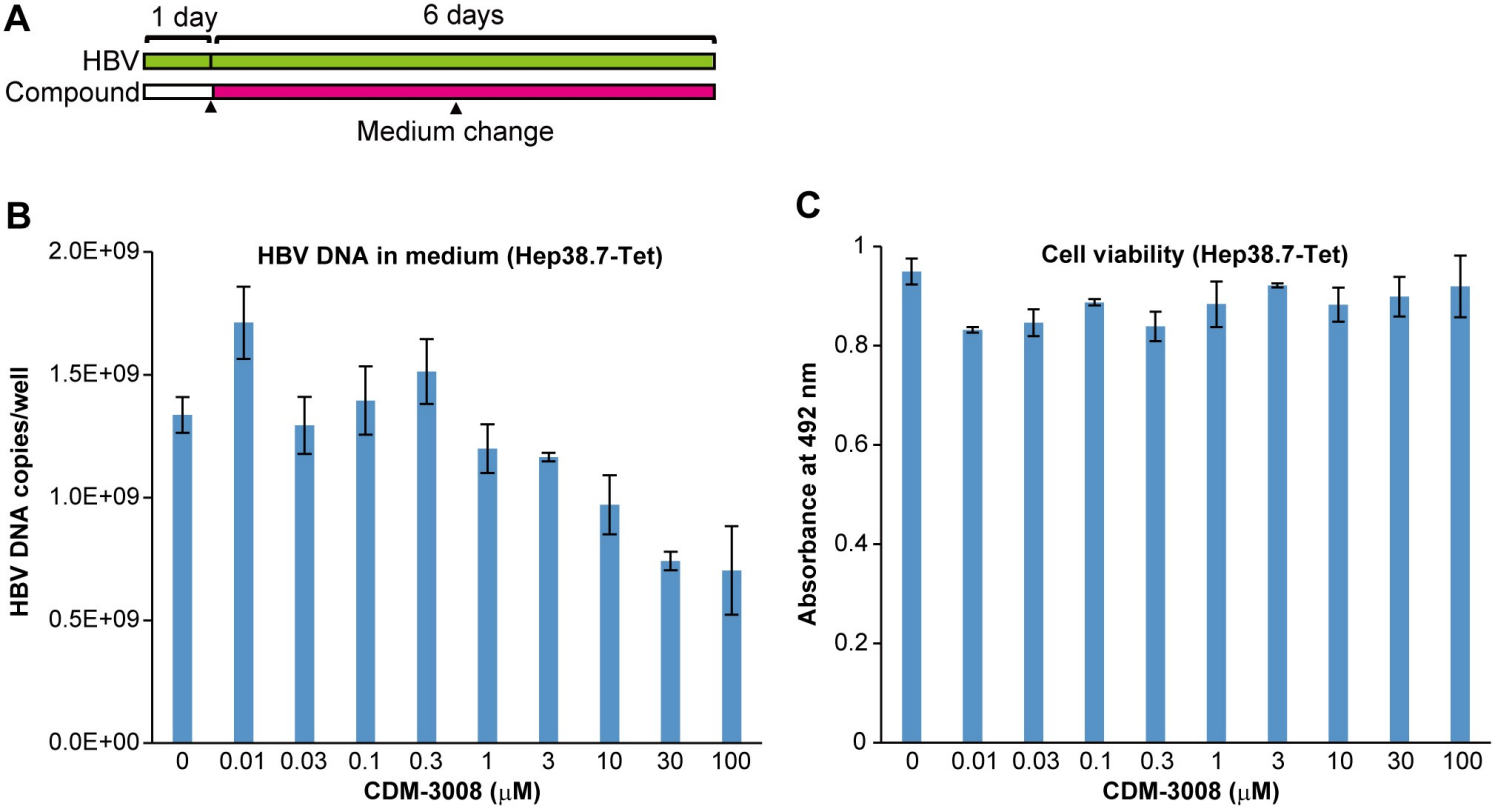
Analysis of anti-HBV activity in HepG2-derived cells. (A) Schematic experimental design of the anti-HBV activity analysis. Hep38.7-Tet cells were cultured for 1 day; the cells were then treated with 0–100 μM CDM-3008 for 6 days (magenta). The black triangles show the times at which the medium was changed. (B, C) Hep38.7-Tet cells were treated with serially diluted CDM-3008 (0–100 μM) for 6 days. After 6 days of treatment, the HBV DNA levels in the cell culture medium were measured by qPCR (B), and cell viability was measured by XTT assay (C). The error bars in B, C indicate S.D. (n = 3).

To confirm that CDM-3008’s anti-HBV activity was mediated mainly through ISGs and not through inhibition of HBV replication, anti-HBV activity was measured in Hep38.7-Tet cells [26]. Because HepG2-derived cells are relatively defective in ISGs expression, IFN does not suppress HBV replication in Hep2.2.15 and HepAD38 cells [27,28]. The IC_50_ of CDM-3008-induced anti-HBV activity in Hep38.7-Tet cells was more than 100 μM and compound did not show cytotoxicity (Fig 3A–3C). The IC_50_ in Hep38.7-Tet cells was at least 100 times higher than the IC_50_ in PXB cells. Thus, we speculated that the anti-HBV activity of CDM-3008 was mainly due to the induced anti-virus activity of ISGs.

**Fig 4:**
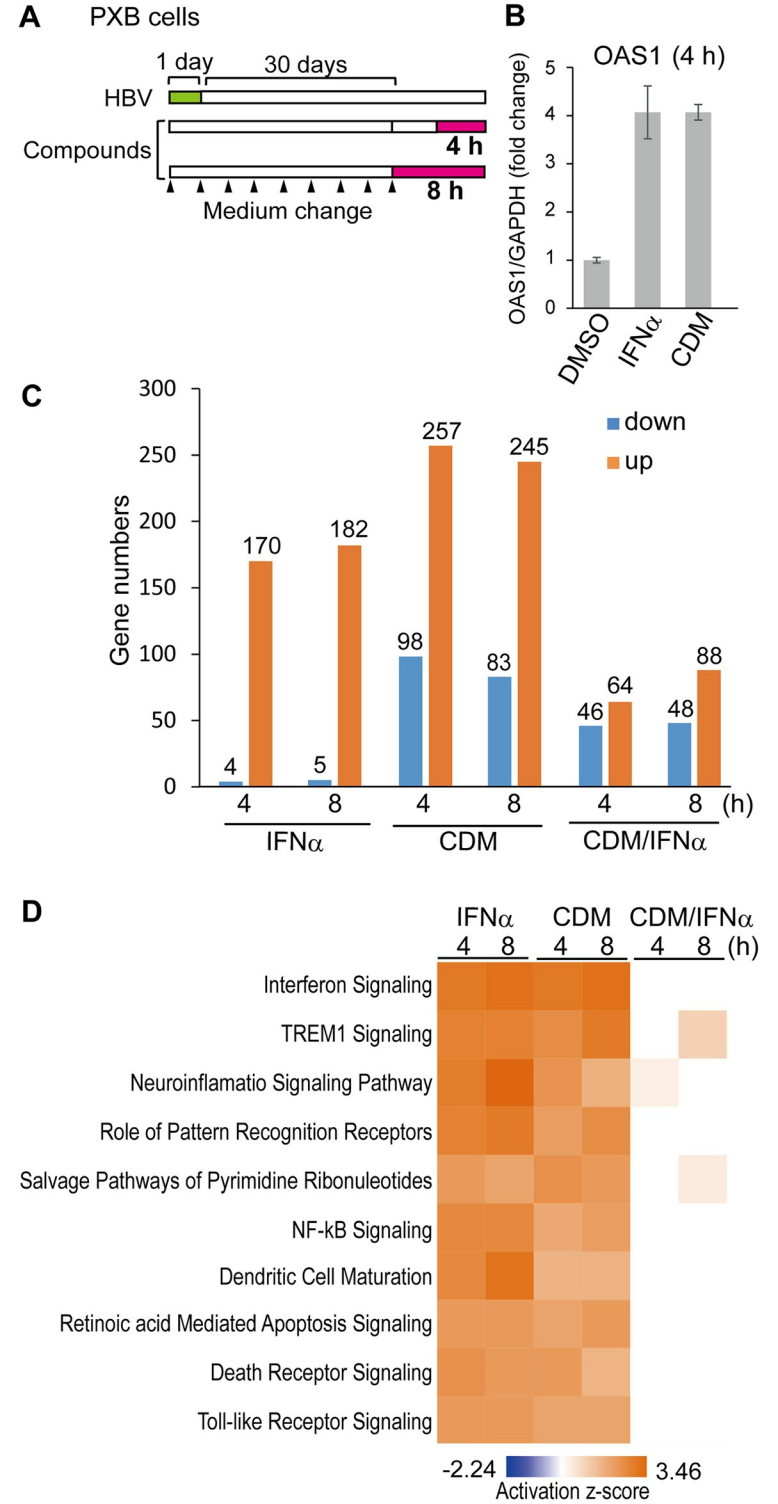
Microarray analysis of mRNA expression after CDM-3008 treatment. (A) Schematic of the experimental design of HBV infection and IFNα and CDM-3008 treatment prior to microarray analysis. PXB cells were infected with HBV genotype C for 1 day (green) and cultured for 30 days. The cells were then treated with 10 ng/ml IFNα or 30 μM CDM-3008 for 4 h or 8 h (magenta). The black triangles show the times at which the medium was changed. (B) Analysis of OAS1 mRNA expression after 4 h of CDM-3008 or IFNα treatment. After 4 h of treatments with 10 ng/ml IFNα or 30 μM CDM-3008, similar levels of OAS1 mRNA were induced. The error bars indicate S.D. (n = 3). (C) Upregulated and downregulated genes. The number of genes whose expression levels were upregulated (orange) or downregulated (blue) by more than 1.2-fold and for which the P-values determined in the microarray analysis were less than 0.001 is shown (n = 3). (D) The top 10 canonical pathways most significantly activated (orange) or inactivated (blue) by IFNα and CDM-3008 were analyzed using the IPA program. The activation z-scores are shown as a heat map.

To determine the molecular mechanism of the anti-HBV activity of CDM-3008, PXB cells were infected with HBV genotype C and cultured for 30 days, and the cells were then treated with 30 μM CDM-3008 or 10 ng/ml IFNα for 4 h and 8 h (Fig 4A). The concentrations of CDM-3008 and IFNα for microarray analysis in PXB cells were shown by qPCR to induce the same expression levels of OAS1 mRNA, which is most abundantly induced by CDM-3008 treatment within ISGs in HCV replicon cells [21], at 4 h of treatments (Fig 4B). Total RNA was purified from the cells and mRNA expression levels were comprehensively analyzed by microarray analysis. Genes that were significantly upregulated or downregulated more than 1.2-fold in CDM-3008 and IFNα-treated cells compared to DMSO-treated cells were quantified (Fig 4C). Within 4 h of IFNα treatment, 4 and 170 genes were downregulated and upregulated, respectively. Within 8 h of IFNα treatment, 5 and 182 genes were downregulated and upregulated, respectively. Within 4 h of CDM-3008-treatment, 98 and 257 genes were downregulated and upregulated, respectively. Within 8 h of CDM-3008-treatment, 83 and 245 genes were downregulated and upregulated, respectively. At 4 h, 46 and 64 genes were significantly upregulated and downregulated more than 1.2-fold, respectively, in CDM-3008-treated cells compared to IFNα-treated cells; at 8 h, 48 and 88 genes were upregulated and downregulated, respectively (Fig 4C). These results suggest that number of genes induced by CDM-3008 was higher than the number induced by IFNα at 4 h and 8 h. Genes whose expression was upregulated or downregulated by more than 1.2-fold in HBV-infected cells compared to non-infected cells were quantified. No genes were upregulated or downregulated after 4 h or 8 h of control (DMSO) treatment. The top 10 canonical pathways that were activated by IFNα and CDM-3008 using the IPA program revealed that cellular signaling pathways were similarly responsive to both treatments; the activation levels of the interferon signaling pathway were particularly similar (Fig 4D).

**Fig 5:**
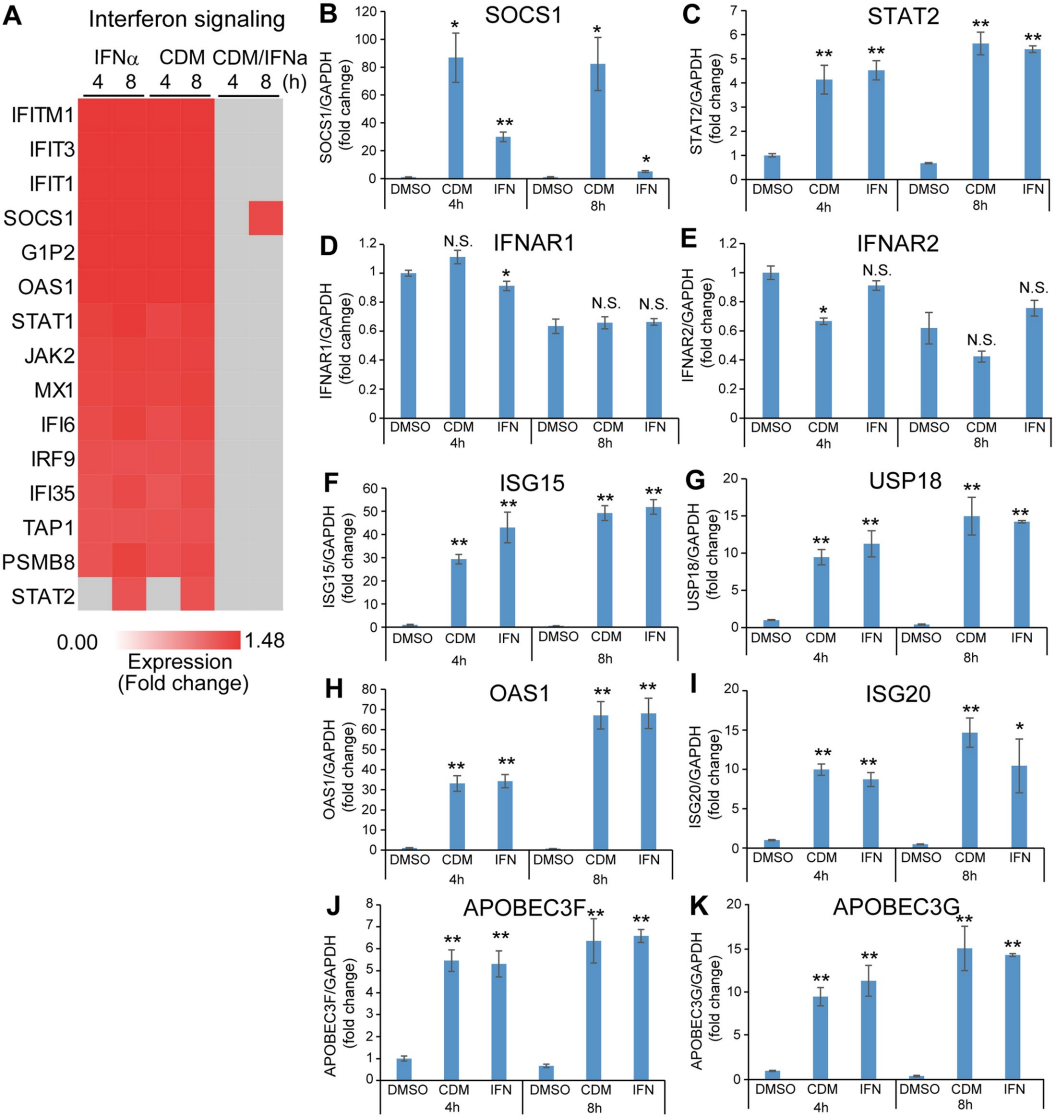
Comparison of molecules involved in the IFN signaling pathway. (A) Heat map showing genes in the interferon signaling pathway that were significantly upregulated by more than 1.2-fold (red) and genes that were significantly upregulated by less than 1.2-fold (gray) genes in the interferon signaling pathway. (B-K) Expression levels of genes associated with interferon signaling. The mRNA expression levels of SOCS1 (B), STAT2 (C), IFNAR1 (D), IFNAR2 (E), ISG15 (F), USP18 (G), OAS1(H), ISG20 (I), APOBEC3F (J), and APOBEC3G (K) were compared at 4 h and 8 h of treatments. The error bars in B-K indicate S.D. (n = 3). *, p < 0.05; **, p < 0.01, N.S., not significant (two-tailed *t*-test) in B-K.

To focus on the differences between IFNα and CDM-3008 in interferon signaling, the expression ratios of genes in the interferon signaling pathway were analyzed using the IPA program. The expression ratios at 4 h and 8 h of treatment with IFNα and CDM-3008 were not significantly different for 14 of the 15 differentially expressed genes shown in the heat map (Fig 5A). However, suppressor of cytokine signaling 1 (SOCS1), which inhibits STAT activation, was upregulated by CDM-3008-treatment at 8 h (Fig 5A), suggesting that feedback inhibition of interferon signaling is strongly induced by CDM-3008 treatment compared with IFNα treatment. To analyze the feedback mechanism and anti-HBV activity of CDM-3008, the expression levels of individual genes in DMSO-, IFNα-, and CDM-3008-treated cells after 4 h and 8 h of treatment were compared by qPCR (Fig 5B–5K). SOCS1 showed greater upregulation with CDM-3008 treatment at 4 h and 8 h than with IFNα treatment (Fig 5B). STAT2 was upregulated by treatment with IFNα and CDM-3008 for 4 h and 8 h compared to DMSO treatment (Fig 5C). IFNAR1 was not downregulated by CDM-3008 treatment, while IFNAR2 was downregulated with CDM-3008-treatment at 4 h (Fig 5D and 5E), suggesting feedback inhibition of the receptor for CDM-3008. ISG15 and UPS18, which form a complex that shuts off downstream signaling of the type I interferon receptor, were upregulated after IFNα and CDM-3008 treatments compared to DMSO treatment (Fig 5F and 5G). 2'-5'-oligoadenylate synthetase 1 (OAS1), which mediates pgRNA degradation through RNase L activity, and ISG20, which degrades RNA/DNA complexes derived from HBV, were upregulated by IFNα and CDM-3008 treatments compared to DMSO treatment (Fig 5H and 5I). Changes in the expression levels of APOBEC3 family genes, including APOBEC3F and 3G, both of which are known to lead to cccDNA degradation, are shown in Fig 5J and 5K. APOBEC3F and 3G were upregulated after IFNα and CDM-3008 treatments (Fig 5J and 5K), whereas no expression of APOBEC3A and 3C in PXB cells was detected by PCR. These results suggest that cccDNA can be degraded by CDM-3008 at least in part through a mechanism similar to the mechanism associated with IFNα.

**Fig 6:**
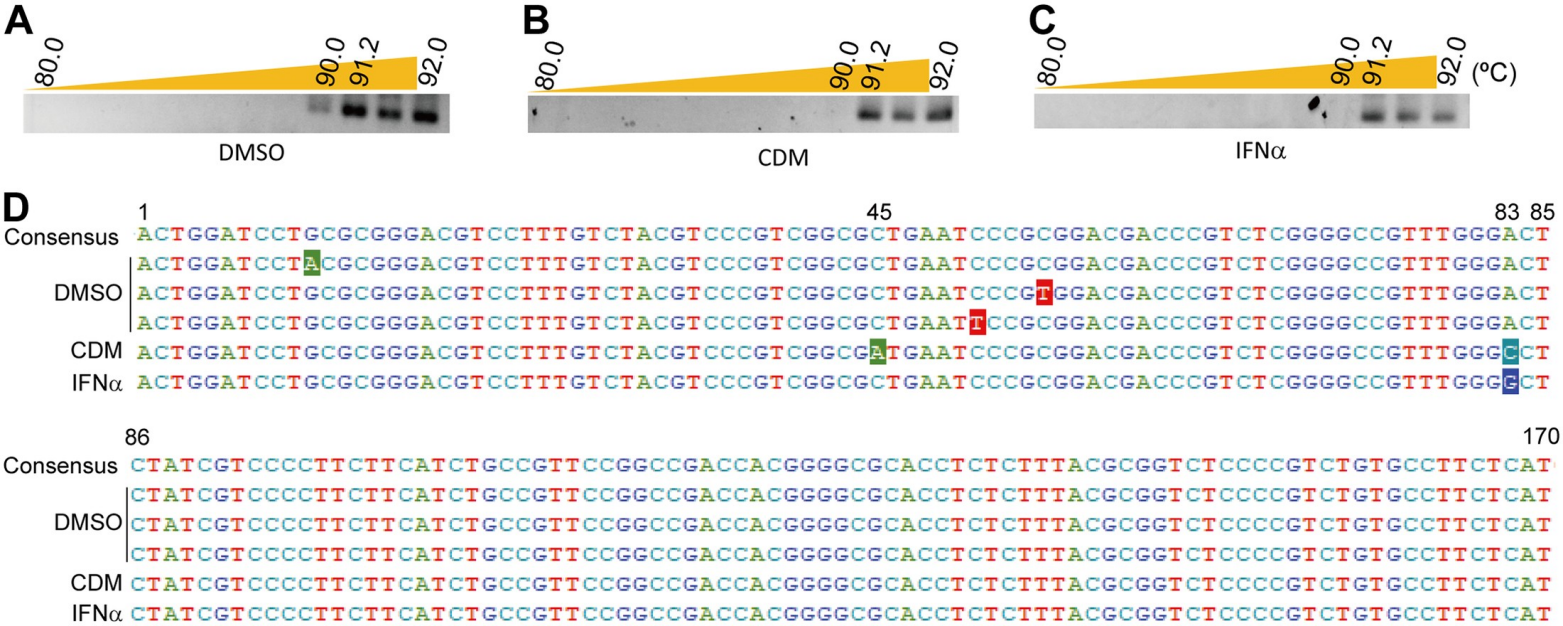
3D-PCR analysis for detection of APOBEC3 activity. (A-C) 3D-PCR products (170 bp) amplified from DMSO- (A), CDM-3008- (B), and IFNα- (C) treated PXB cells at different denaturation temperatures (80–92 °C) were separated by 2% agarose gel electrophoresis and visualized. (D) The 3D-PCR products obtained at a denaturing temperature of 91.2 °C were sequenced, aligned with the consensus sequence, and numbered. Mutations are shown as inverted letters.

To show the molecular mechanism of anti-HBV activity induced by CDM-3008, we performed 3D-PCR for detection of APOBEC3 activity. PXB cells were infected with HBV of genotype C for 1day and cultured for 28 days; the cells were then treated with 100 μM CDM-3008 or 10,000 IU/ml IFNα for 7 days. After purification of DNA from the cells, cccDNA was first amplified by PCR and then the HBx region was amplified by nested PCR (Fig 6A–6C). A shift to a lower denaturing temperature was only detected in DMSO-treated PXB cells but not in CDM-3008- or IFNα-treated PXB cells. The PCR products of the HBx region (170 bp) at a denaturation temperature of 91.2 °C were cloned and sequenced (Fig 6D). Approximately 30 clones were sequenced from each conditions. In the DMSO-treated samples, C to T and G to A transitions at random positions were observed in 21.7% of the clones. In contrast, C to A transition at position 45 and A to C transition at position 83 were observed in 26.9% of the clones in the CDM-3008-treated samples, and A to G transition at position 83 was observed in 29.6% of the clones in the IFNα-treated samples. These results suggest that cccDNA molecules that have undergone random transitions due to APOBEC3 deaminase activity have already been degraded in CDM-3008- and IFNα-treated PXB cells.

We selected genes that were specifically induced by CDM-3008-treatment, but not by IFNα. The top 50 most highly expressed genes are shown in Table 1. Serine dehydratase (SDS), suppressor of cytokine signaling 3 (SOCS3), chromosome 11 open reading frame 96 (C11orf96), serum amyloid A2 (SAA2), and cytokine inducible SH2 containing protein (CISH) were the most abundantly and specifically induced by CDM-3008-treatment. SOCS3 and CISH are family proteins that inhibit STAT activation [29], and SOCS1 was highly induced by CDM-3008 compared to IFNα (Fig 5B), suggesting that CDM-3008 efficiently induces feedback inhibition of the interferon signaling pathway.

**Table 1:** Top 50 genes specifically upregulated by CDM-3008 treatment.

					Average	
Rank	Gene ID	Symbol	Entrez Gene Name	Type(s)	IFN /DMSO	CDM /DMSO
1	10993	SDS	serine dehydratase	enzyme	1.02	2.18
2	9021	SOCS3	suppressor of cytokine signaling 3	phosphatase	1.03	2.14
3	387763	C11orf96	chromosome 11 open reading frame 96	other	0.95	2.06
4	6289	SAA2	serum amyloid A2	other	1.05	1.95
5	1154	CISH	cytokine inducible SH2 containing protein	other	0.98	1.93
6	5596	MAPK4	mitogen-activated protein kinase 4	kinase	1.10	1.75
7	1723	DHODH	dihydroorotate dehydrogenase (quinone)	enzyme	0.97	1.73
8	5320	PLA2G2A	phospholipase A2 group IIA	enzyme	1.02	1.72
9	5105	PCK1	phosphoenolpyruvate carboxykinase 1	kinase	1.14	1.69
10	6288	SAA1	serum amyloid A1	transporter	1.02	1.64
11	3934	LCN2	lipocalin 2	transporter	1.03	1.64
12	9214	FAIM3	Fc fragment of IgM receptor	other	1.02	1.61
13	644945	KRT16P3	keratin 16 pseudogene 3	other	1.08	1.55
14	5292	PIM1	Pim-1 proto-oncogene, serine/threonine kinase	kinase	0.93	1.49
15	10344	CCL26	C-C motif chemokine ligand 26	cytokine	1.00	1.48
16	9510	ADAMTS1	ADAMTS type 1 motif 1	peptidase	0.98	1.48
17	4837	NNMT	nicotinamide N-methyltransferase	enzyme	1.04	1.47
18	11238	CA5B	carbonic anhydrase 5B	enzyme	1.02	1.47
19	14415	GAD1	glutamate decarboxylase 1	enzyme	1.11	1.45
20	220108	FAM124A	family with sequence similarity 124 member A	other	1.11	1.45
21	2243	FGA	fibrinogen alpha chain	other	1.08	1.45
22	7004	TEAD4	TEA domain transcription factor 4	transcription	1.11	1.43
23	3929	LBP	lipopolysaccharide binding protein	transporter	1.01	1.43
24	8740	TNFSF14	TNF superfamily member 14	cytokine	0.91	1.42
25	79689	STEAP4	STEAP4 metalloreductase	enzyme	1.02	1.42
26	10406	WFDC2	WAP four-disulfide core domain 2	other	1.03	1.42
27	10912	GADD45G	growth arrest and DNA damage inducible γ	other	0.96	1.41
28	23151	GRAMD4	GRAM domain containing 4	other	0.97	1.41
29	1116	CHI3L1	chitinase 3 like 1	enzyme	0.99	1.41
30	7043	TGFB3	transforming growth factor beta 3	growth factor	1.00	1.41
31	27289	RND1	Rho family GTPase 1	enzyme	0.96	1.40
32	283777	FAM169B	family with sequence similarity 169 member B	other	1.00	1.39
33	6446	SGK1	serum/glucocorticoid regulated kinase 1	kinase	1.01	1.38
34	57509	MTUS1	microtubule associated scaffold protein 1	other	0.98	1.38
35	53905	DUOX1	dual oxidase 1	enzyme	1.01	1.38
36	7504	NA	X-linked Kx blood group	transporter	1.00	1.38
37	642273	FAM110C	family with sequence similarity 110 member C	other	1.08	1.37
38	10507	SEMA4D	semaphorin 4D	receptor	1.12	1.37
39	8829	NRP1	neuropilin 1	receptor	1.09	1.37
40	3726	JUNB	JunB proto-oncogene, AP-1 transcription factor subunit	transcription	1.11	1.36
41	3283	HSD3B1	hydroxy-δ-5-steroid dehydrogenase, 3β- and steroid δ-isomerase 1	enzyme	0.99	1.36
42	6590	SLPI	secretory leukocyte peptidase inhibitor	other	1.07	1.36
43	3394	IRF8	interferon regulatory factor 8	transcription	1.08	1.36
44	10957	PNRC1	proline rich nuclear receptor coactivator 1	other	1.06	1.36
45	388125	C2CD4B	C2 calcium dependent domain containing 4B	other	0.98	1.36
46	89870	TRIM15	tripartite motif containing 15	other	0.97	1.35
47	6347	CCL2	C-C motif chemokine ligand 2	cytokine	1.09	1.35
48	10509	SEMA4B	semaphorin 4B	other	1.03	1.34
49	64856	VWA1	von Willebrand factor A domain containing 1	other	0.99	1.34
50	116372	LYPD1	LY6/PLAUR domain containing 1	GPCR	0.97	1.34

The gene ID, symbol, Entrez gene name, type of gene, and average expression level are indicated in the table. Expression levels of genes in IFNα- and CDM-3008-treated cells were divided by the expression levels of genes in DMSO-treated cells. Genes that were specifically upregulated in CDM-3008-treated cells and not in IFNα-treated cells were selected, and the top 50 most highly expressed genes are shown.

**Fig 7:**
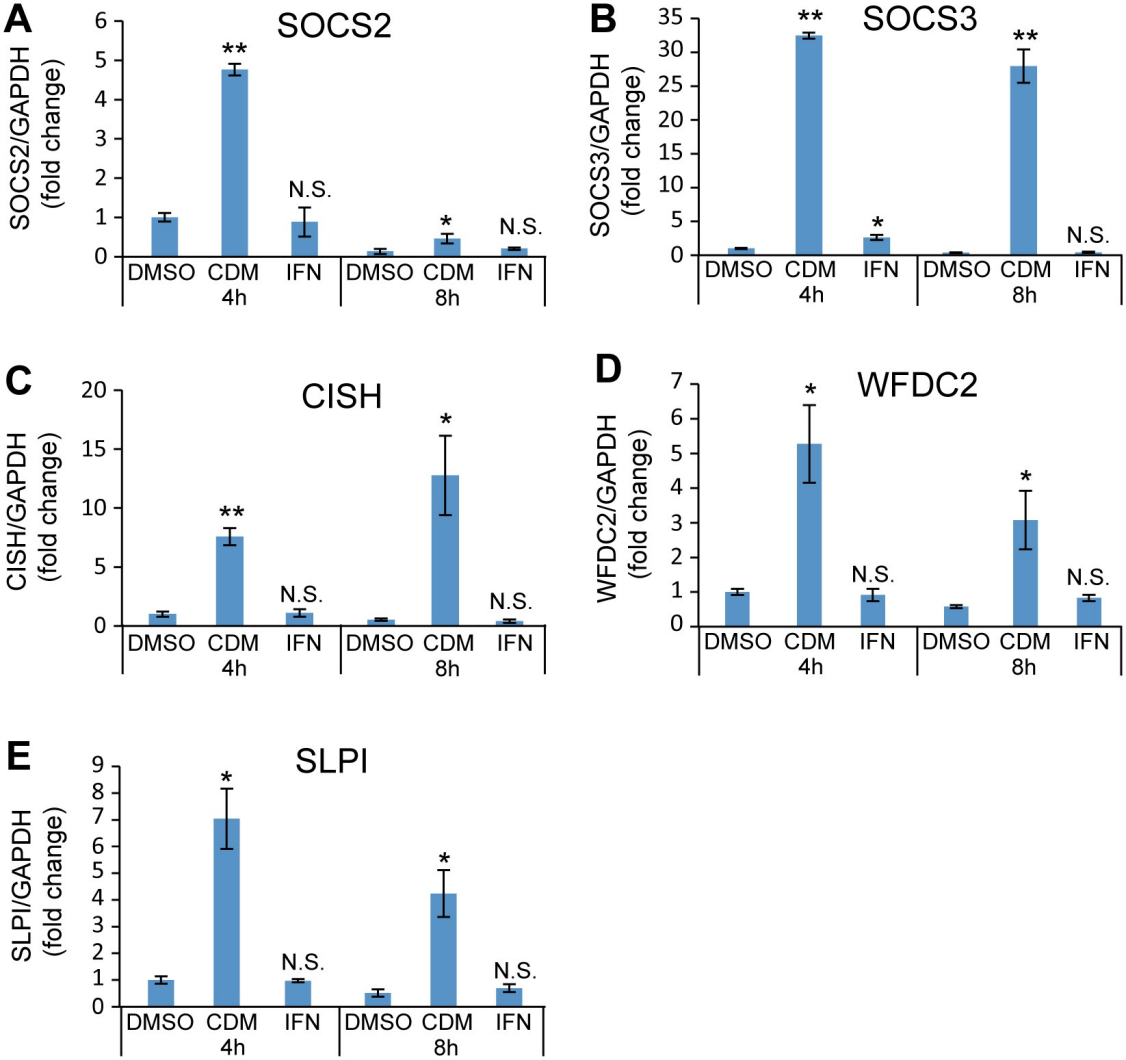
qPCR analysis of CDM-3008-specific genes. (A-E) mRNA expression levels of SOCS2 (A), SOCS3 (B), CISH (C), WFDC2 (D), and SLPI (E) were compared at 4 h and 8 h of treatments. The error bars in A-E indicate S.D. (n = 3). *, p < 0.05; **, p < 0.01; N.S., not significant (two-tailed *t*-test) in A-E.

CDM-3008-mediated specific expression of SOCS2, SOCS3, CISH, WFDC2, and SLPI was confirmed by qPCR (Fig 7A–7E).

**Fig 8:**
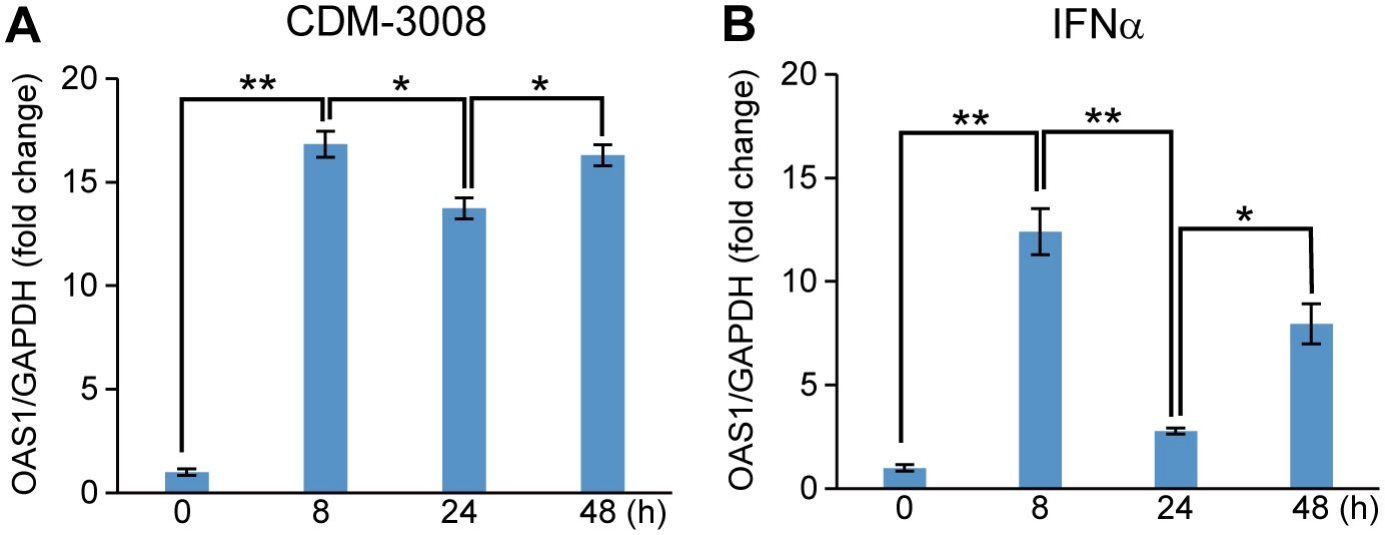
Time course of OAS1 expression after CDM-3008 or IFNα treatment. (A-B) OAS1 mRNA expression levels were compared after 0, 8, 24 and 48 h of CDM-3008 (A) and IFNα (B) treatment. The error bars in A, B indicate S.D. (n = 3). *, p < 0.05; **, p < 0.01 (two-tailed *t*-test) in A, B.

To analyze feedback inhibition of STAT signaling, the time-dependent expression of OAS1 was analyzed in CDM-3008-treaetd (Fig 8A) and IFNα-treated (Fig 8B) PXB cells. PXB cells were treated with 30 μM CDM-3008 or 10 ng/ml IFNα for 0, 8, 24, and 48 h. OAS1 expression levels in CDM-3008 and IFNα-treated cells were increased at 8 h, then decreased at 24 h, and increased at 48 h (Fig 8A and 8B).

**Fig 9:**
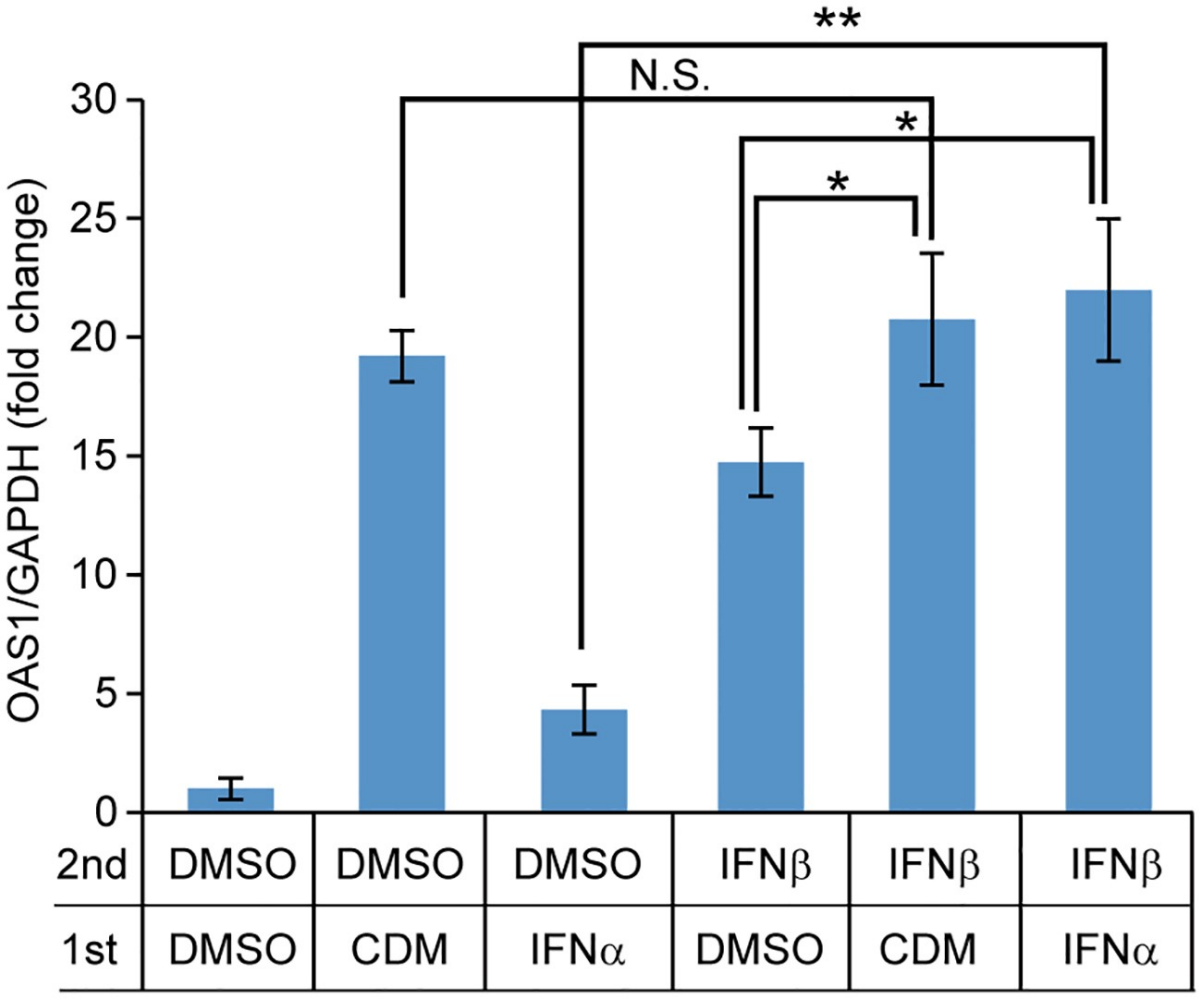
OAS1 expression of IFNβ-treated cells pre-incubated with CDM-3008 or IFNα. PXB cells were pretreated with 30 μM CDM-3008 (CDM) or 10 ng/ml IFNα for 24 h and then treated with 10 ng/ml IFNβ for 4 h. mRNA expression levels of OAS1 were compared. The error bars indicate S.D. (n = 3). *, p < 0.05; **, p < 0.01; N.S., not significant (two-tailed *t*-test).

Makowska et al. reported that repeated administration of IFNβ and IFNλ activates the JAK/STAT pathway without showing feedback inhibition [30]. To show feedback inhibition of IFN-signaling induced by CDM-3008, PXB cells were stimulated by IFNβ after pretreatment with CDM-3008 or IFNα. OAS1 expression induced by CDM-3008 was not significantly different with or without IFNβ treatment, whereas OAS1 expression induced by IFNα was increased by IFNβ treatment (Fig 9). Thus, CDM-3008 induced stronger feedback inhibition of ISGs expression than IFNα.

**Fig 10:**
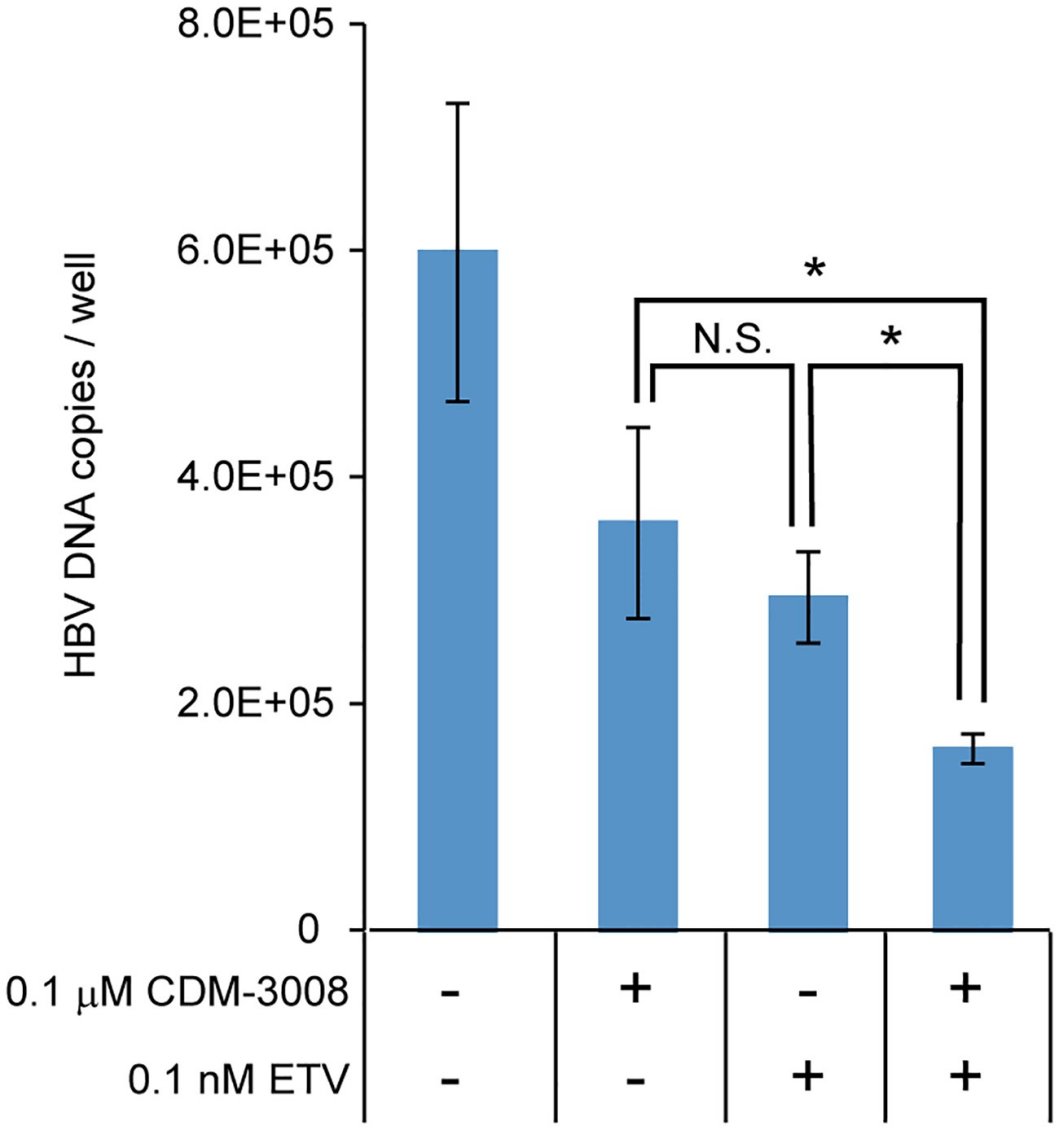
Additive effect of CDM-3008 and entecavir on anti-HBV activity. Primary cultured human hepatocytes (PXB cells) were infected with HBV genotype C for 1day and cultured for 28 days; the cells were then treated with 0.1 μM CDM-3008 and/or 0.1 nM entecavir (ETV) for 7 days. HBV DNA copies in PXB cells were measured by qPCR. The error bars indicate S.D. (n = 3). *, p < 0.05 (two-tailed *t*-test).

To test the additive effect of CDM-3008 and entecavir (ETV), anti-HBV activity was analyzed in PXB cells that had been treated with 0.1 μM CDM-3008 and/or 0.1 nM ETV (Fig 10). The concentrations of CDM-3008 and ETV used in the experiment were selected based on the IC_50_ of the compounds for anti-HBV activity in PXB cells. Treatment with a combination of CDM-3008 and ETV significantly reduced the levels of HBV DNA compared with single treatment of CDM-3008 or ETV. Thus, we concluded that CDM-3008 and ETV have an additive effect.

## Discussion

In this study, we show that CDM-3008 had similar anti-HBV activity to IFNα mainly through ISGs induction. Based on the expression analysis, anti-HBV activity was induced by the RNase L activity associated with OAS1, the exonuclease activity of ISG20 for degradation of RNA-DNA complexes, and the deaminase activity of APOBEC3 family proteins for cccDNA degradation [16,31,32].

CDM-3008 induced higher levels of genes involved in feedback inhibition such as SOCS1, SOCS2, SOCS3, and CISH than IFNα [29]. CDM-3008 suppresses IFNAR2 expression. CDM-3008 and IFNα induced similar expression levels of ISG15 and USP18 [33]. Thus, CDM-3008 can effectively inhibit interferon signaling by suppressing its receptor and downstream signaling.

Several acute phase proteins were involved in CDM-3008-induced genes such as SAA1, SAA2, WFDC2, and SLPI, as shown in Table 1. SAA1 and SAA2 expression levels are increased with infection, inflammation, and injury. SAA has cytokine-like activity in the inflammatory response and induces cytokines and chemokines. SAA is highly expressed in HCC associated with HBV [34]. Thus, SAA1 and SAA2 may contribute to inflammation and innate immunity resulting from HBV.

Anti-viral peptides are enhanced in their expression and secreted into the extracellular space in response to acute inflammation. WFDC2 and SLPI were upregulated by CDM-3008-treatment. WFDC2 and SLPI have a whey acidic protein (WAP) motif. WAP motif-containing peptides are part of a family containing WFDC2, SLPI, and elafin and are often associated with anti-viral and anti-bacterial activities [35,36]. Elafin is secreted form epithelial cells and shows anti-HIV-1 activity [37,38]. WAP-motif containing peptides are possible anti-HBV factors in the innate immune system.

In this study, the donor of the hepatocytes used in the assays was changed after the microarray analysis was conducted. This occurred because the supplier of the hepatocytes, PhoenixBio Inc., prepares human liver chimeric mice from new donors approximately every 6 years. The expression levels of ISGs after CDM-3008 and IFNα treatment differed depending on the donor of the hepatocytes. Fig 4B shows that OAS1 expression levels in CDM-3008- and IFNα-treated cells were 4 times higher than the levels in control DMSO-treated cells when an old donor of hepatocytes was used, while Fig 5H shows that OAS1 expression in CDM-3008- and IFNα-treated cells was approximately 30 times higher than in control DMSO-treated cells when a new donor of hepatocytes was used. The response to IFNα and ribavirin in hepatitis C virus-infected patients is associated with a single-nucleotide polymorphism in the IL28 gene [39]. Thus, the response to CDM-3008 and IFNα appears to different depending on genetic variation in the donor.

Niu et al. reported that established HBV infection does not significantly modulate the transcriptional response to various innate immune stimuli [40]. In this study, difference more than 1.2-fold in the expression levels of host factors was not observed when HBV-infected and non-infected PXB cells were analyzed by microarray. In our experiments, PXB cells were infected with 3 genome equivalents of HBV and cultured for 30 days. The number of HBV copies/cell was calculated to be approximately 1 copy/cell (Fig 1). It is expected that 1 copy of HBV per hepatocyte would have little effect on host factors. In addition, PXB cells were cultured for 30 days after infection with HBV; the innate immune response would be expected to occur during the early stage of infection and to decrease during the 30 days following infection.

CDM-3008 treatment of PXB cells suppressed cccDNA and HBeAg, which is thought to be a marker for cccDNA (Fig 1) [41,42]. APOBEC3F and 3G were highly expressed in PXB cells after CDM-3008 treatment. Thus, cccDNA could be degraded due to the deaminase activity of APOBEC3F and 3G. To confirm APOBEC3 activity, we performed 3D-PCR. Random C to A and G to T transitions were detected in DMSO-treated PXB cells, while C to A and A to C transitions at positions 45 and 83 and A to G transition at position 83 were detected in CDM-3008- and IFNα-treated PXB cells, respectively (Fig 6D). These results suggest that cccDNA molecules that have undergone random transitions due to APOBEC3 deaminase activity have already been degraded in CDM-3008- and IFNα-treated PXB cells after exposure of the cells to the treatment for 7 days. Kitamura et al. showed that transitions induced by APOBEC3G were mainly G to A transitions [43]. The transitions at position 83 induced by CDM-3008 and IFNα appear to differ from the transitions induced by APOBEC3 family proteins, indicating that the other modifying enzymes specifically induced by CDM-3008 and IFNα could be involved in cccDNA modification.

Both CDM-3008 and IFNα exert their anti-HBV activity primarily through activation of the JAK/STAT pathway and ISGs induction. CDM-3008 is a small chemical compound that is available for oral administration and can be produced at low cost, indicating an advantage of CDM-3008 over IFNα [21]. Orally administered CDM-3008 is thought to be absorbed from the intestine and delivered to the liver through the portal vein. Thus, CDM-3008 could be efficiently delivered to the liver, reducing side effects compared to intravenously injected IFNα. Orally administered CDM-3008 can be used with the other newly developing orally administered drugs, such as nucleotide analogues and capsid allosteric modulators. CDM-3008 induced ISGs expression only through IFNAR2, while IFNα induced ISGs expression through a heterodimer of IFNAR1 and IFNAR2 (Fig 2). Thus, IFNAR1 downstream signaling associated with side effects could be suppressed. CDM-3008 showed relatively higher feedback inhibition of JAK/STAT signaling than IFNα and easily controlled JAK/STAT activity. This feature may suggest an approach to regulating side effects. However, CDM-3008 has problems with solubility and metabolic stability that may affect its use as a drug. We have initiated a structure-activity relationship study to improve the solubility of CDM-3008. CDM-3032, a derivative of CDM-3008, shows improved solubility and metabolic stability in both mouse and human hepatic microsomes [44]. We are continuing a further structure-activity relationship study of CDM-3008 with a goal of improving its solubility, metabolic stability, and anti-HBV activity and reducing its potential side effects associated with IFNα.

## Conclusions

CDM-3008 is a small chemical compound that exerts anti-HBV activity mainly through the interferon signaling pathway; its activity includes induction of the expression of ISGs and APOBEC3 family proteins. Feedback inhibition of interferon signaling induced by CDM-3008 appears to be stronger than that induced by IFNα. CDM-3008 is potentially available for oral administration and cotreatment with nucleotide analogs and can be produced at a low cost, indicating advantages of CDM-3008 over IFNα.
